# Identifying primary care clinicians’ preferences for, barriers to, and facilitators of information-seeking in clinical practice in Singapore: a qualitative study

**DOI:** 10.1186/s12875-024-02429-x

**Published:** 2024-05-18

**Authors:** Mauricette Moling Lee, Wern Ee Tang, Helen Elizabeth Smith, Lorainne Tudor Car

**Affiliations:** 1 Lee Kong Chian School of Medicine, Nanyang Technological University, Singapore, Novena Campus Clinical Sciences Building 11 Mandalay Road, Singapore, 308232 Singapore; 2 Singapore Institute of Technology, 10 Dover Drive, Singapore, 138683 Singapore; 3 Clinical Research Unit, National Health Group Polyclinics (HQ), 3 Fusionopolis Link, Nexus @ One-North, Singapore, 138543 Singapore; 4 Family Medicine and Primary Care, Lee Kong Chian School of Medicine, Nanyang Technological University, Singapore, Novena Campus Clinical Sciences, Building 11 Mandalay Road, Singapore, 308232 Singapore; 5 Department of Primary Care and Public Health, School of Public Health, Imperial College London, London, UK

**Keywords:** Evidence-based medicine, Information-seeking behaviour, Nurses, Physicians, Smartphone

## Abstract

**Background:**

The growth of medical knowledge and patient care complexity calls for improved clinician access to evidence-based resources. This study aimed to explore the primary care clinicians’ preferences for, barriers to, and facilitators of information-seeking in clinical practice in Singapore.

**Methods:**

A convenience sample of ten doctors and ten nurses was recruited. We conducted semi-structured face-to-face in-depth interviews. The interviews were recorded, transcribed verbatim, and analysed using thematic content analysis.

**Results:**

Of the 20 participants, eight doctors and ten nurses worked at government-funded polyclinics and two doctors worked in private practice. Most clinicians sought clinical information daily at the point-of-care. The most searched-for information by clinicians in practice was less common conditions. Clinicians preferred evidence-based resources such as clinical practice guidelines and UpToDate®. Clinical practice guidelines were mostly used when they were updated or based on memory. Clinicians also commonly sought answers from their peers. Furthermore, clinicians frequently use smartphones to access the Google search engine and UpToDate® app. The barriers to accessing clinical information included the lack of time, internet surfing separation of work computers, limited search functions in the organisation’s server, and limited access to medical literature databases. The facilitators of accessing clinical information included convenience, easy access, and trustworthiness of information sources.

**Conclusion:**

Most primary care clinicians in our study sought clinical information at the point-of-care daily and reported increasing use of smartphones for information-seeking. Future research focusing on interventions to improve access to credible clinical information for primary care clinicians at the point-of-care is recommended.

**Trial registration:**

This study has been reviewed by NHG Domain Specific Review Board (NHG DSRB) (the central ethics committee) for ethics approval. NHG DSRB Reference Number: 2018/01355 (31/07/2019).

**Supplementary Information:**

The online version contains supplementary material available at 10.1186/s12875-024-02429-x.

## Background

Primary care clinicians provide the bulk of care to patients in primary care settings. In Singapore, there are 23 polyclinics and about 1,800 General Practitioner (GP) clinics with private GPs providing primary care for about 80% of the population [1]. The primary care clinicians provide primary care services at community polyclinics and private medical clinics around Singapore [1]. The polyclinics are formed by three healthcare groups – National Healthcare Group, National University Health System, and SingHealth [1]. These polyclinics served various populations in Singapore's central, northern, north-eastern, western, and eastern parts [1]. Every day, clinicians make many clinical decisions, ranging from diagnosis and prognosis to treatment and patient management [2, 3]. However, to provide consistent high-quality patient care, such clinical judgments must be informed by existing trustworthy medical evidence [4–6]. To meet their information needs, clinicians seek relevant information from various sources of information [3]. Searching for and using the information to meet information needs has been described as information-seeking behaviour [7–9].

Previous research showed that clinicians often raise questions about patient care in their practice [10]. Half of those questions are left unanswered. Identifying what information primary care clinicians need, how they search for required information and how they adopt it into practice is essential in ensuring safe and high-quality patient care [11, 12]. While there are reports of information-seeking behaviour in primary care from other countries [2, 8, 13, 14], similar reports in Singapore are limited.

Clinicians may consult several sources to support their decisions, including clinical practice guidelines (CPGs), journal articles, peers, and more [3]. However, there is a wide variation in the adoption of evidence-based practices across healthcare disciplines, which could lead to poorer primary care outcomes [8, 12, 15–19]. To mitigate this, a commonly employed approach is the development of CPGs, clinical pathways, or care guides [20]. They offer a structured, reliable, and consistent approach to healthcare evidence dissemination and reduce unnecessary clinical practice variation [21]. However, CPGs are costly to develop and update, context-specific, and unevenly adopted across various healthcare systems [22]. CPG's uptake is affected by diverse factors such as presentation formats, time pressures, reputability, and ownership [14, 23]. Conversely, other sources of clinical practice-related information may not be as valid, credible, or current as CPGs.

Increasingly, healthcare professionals worldwide use their smartphones as an important channel for clinical information [24–27], using them to access websites, mobile apps or communicate with peers [28]. The use of electronic resources improves clinicians' knowledge and behaviour as well as patients' outcomes [29]. However, evidence on how smartphones are used at the point-of-care, particularly for evidence-seeking, is limited. Singapore, with a total population of 5.92 million as of the end of June 2023 [30], is one of the countries with the highest smartphone usage among its residents, with approximately 5.72 million (97%) users in 2023 [31]. Correspondingly, smartphones may be an important information-seeking channel among primary care clinicians. However, the increasing cyber threats worldwide may lead to internet surfing separation as a common security measure.

Institutional policies limiting access to computers at the point-of-care deter clinicians from seeking information and disrupt their workflow [32]. Due to patient data privacy breaches, the Singapore Ministry of Health introduced internet surfing separation as a security measure in July 2018 in all public healthcare institutions in Singapore [33]. Internet surfing separation stands for the restrictions on internet access and browsing which were enforced in Singapore public healthcare institutions in 2018 due to patient data privacy breaches [33]. This has limited the internet access of primary care clinicians at the workplace. Since its introduction, the Internet has not been accessible from any of the clinic's desktop computers and has been available through a few work laptops with limited availability to the polyclinic staff. At the time that this research was conducted, primary care clinicians in the public healthcare sector in Singapore did not have access to the internet from their work computers. Clinicians rely on evidence-based information to make informed decisions about patient care [4–6]. When access to online resources is restricted, clinicians may struggle to receive current and correct information, thus jeopardising patient safety and the quality of care offered [11, 12]. Therefore, we sought to understand how primary care clinicians were addressing their clinical information needs when their work computers were not available to access evidence-based resources online. This study aimed to explore the primary care clinicians’ preferences for, barriers to, and facilitators of information-seeking in clinical practice in Singapore.

## Methods

A qualitative study consisting of semi-structured face-to-face in-depth interviews was used to explore the primary care clinicians’ preferences for, barriers to, and facilitators of information-seeking in clinical practice in Singapore. The interviews were conducted between August and November 2019 at two polyclinics and two private clinics in Singapore.

The study was approved by the institutional ethics committee (NHG DSRB Reference Number: 2018/01355). All participants read the study information sheet before providing written consent. This study followed the Consolidated Criteria for Reporting Qualitative Research guidelines [34] [see Additional file 1].

### Participants and recruitment

We included primary care doctors and registered nurses from the polyclinics and private primary care practices aged ≥ 21 years who were fluent in English. We employed convenience sampling in this study. Prospective participants were recruited from various polyclinics through personal contacts and advertisements. Five potential participants were contacted but did not respond to the invitation, two potential participants declined participation in this study and one potential participant resigned before the commencement of the study and hence did not participate in the study.

### Data collection

The interviews were conducted by a female researcher (MML) in designated private meeting rooms or consultation rooms at various polyclinics or the respective consultation rooms of the private practice. MML was provided with sufficient details, resources, and training on qualitative research before the study commencement. Before the start of the interview, the researcher introduced herself, stated the aim of the interview, explained confidentiality, and obtained informed consent and permission to use a digital voice recorder. The interviewees could pause the interviews due to professional responsibilities at any time. MML conducted the interviews using an interview guide based on a review of the relevant literature and team discussions [10] [see Additional file 2]. The interview topics included the type of questions during clinical encounters, commonly employed sources of clinical information, frequency and timing of information-seeking, satisfaction with existing information sources, use of CPGs, barriers to information-seeking, and reliability of obtained information. All interview sessions lasted not more than 60 minutes with a mean interview time of 25 minutes and were digitally recorded and transcribed. Field notes were taken during the interviews for further analysis. Data saturation, defined as no new themes arising after three consecutive interviews [35], was achieved after 20 interviews, therefore we stopped recruitment at 20 participants. Participants were compensated with a SGD25 voucher and a meal upon completion of the interview.

### Data analysis

The qualitative data were analysed using Burnard’s method, a structured approach for thematic content analysis established in 1991 [36]. Burnard's method includes fourteen stages for categorising and coding interview transcripts [36] [see Additional file 4]. Types of questions were analysed using Ely’s classification [37]. Burnard's method enhances understanding of the information-seeking behaviour patterns found by Ely's approach by doing a comprehensive evaluation. Ely et al. (2000) developed an approach for categorising clinician queries about patient care [37]. Clinical questions in primary care were divided into several main categories. For example, the three most common categories of questions based on Ely’s approach were "What is the drug of choice for condition x?", "What is the cause of symptom x?" and "What test is indicated in situation x?" [37]. Ely et al. (2000) framework was used by the study team to gain a better understanding of clinicians' information needs and to identify the types of questions they had about patient care. It was used mainly to facilitate the study team’s discussion. The study team did not adopt the categories. The analysis was done independently and in parallel by two researchers (MML and LTC). First, the researchers familiarised themselves with the transcripts by reading them multiple times. Second, the initial codes were proposed. Third, the themes were derived from the codes. Fourth, the researchers discussed and combined their themes for comparison. Finally, they reached a consensus on the themes and how to define them. Apart from the initial stages of being acquainted with the transcripts and recommended initial codes, to streamline our codes, related codes were consolidated into more comprehensive headings. This process allows us to organise them more effectively under pertinent subthemes. For example, various information sources that were mentioned by the participants such as evidence-based resources, non-evidence-based resources, and colleagues have all been merged into a subtheme titled "popular information sources" [see Additional file 3]. This process was done iteratively through several rounds. The final list of themes and subthemes was created by removing repeated or similar subthemes. Two other study team members independently created a list of headings without using the first study team member's list. Three lists were discussed and improved to increase validity and reduce researcher bias. Finally, we employed abstraction by developing a basic description of the phenomenon under investigation to establish the final subthemes and themes. Tables 1 and 2 illustrate how these stages were conducted.

**Table 1:** Excerpt of coding memo

Transcript	**Codes**	**Notes**
“Even doing a kind of list search to see what are the recommendations…we seldom see in primary care” Doctor01	Rare condition	Most searched information in clinical practice
“Other ones that I would search for would be if the patient comes in with very…unusual presentations.” Doctor07		
“what other investigations I should do for conditions I’m not that familiar with.” Doctor02		
“Conditions, which we…may not be that familiar with offhand.” Doctor04

**Table 2:** Example of thematic content analysis process

Transcript	**Codes**	**Subthemes**	**Themes**
“doing a kind of list search to see what are the recommendations…we seldom see in primary care” Doctor01	Rare condition	The type of information needs	Accessing information sources
“pharmacology, or new medicine-related information, or what’s the standard clinical practice, most of the time all this information if this are all under our regular clinical practice” Doctor01	Pharmacology		
	“New medicine-related information”		
	Standard clinical practice		
“teaching pedagogy skills, sometimes…I will just Google or go to any other relevant websites…to look for information that I want” Doctor01	Teaching pedagogy skills		

Table 1 illustrates that the previous "subtheme" for "rare condition" was "most searched information in clinical practice," but it has been revised to "the type of information needs" to include numerous codes such as pharmacology and others following additional discussion with study team members. A third reviewer HES acted as an arbiter. The coding of transcripts was performed using a word processor. A predetermined classification system was not employed since there was insufficient research to inform the clinicians' perceptions of information-seeking behaviour in Singapore. In particular, the dynamic identification of themes from data was facilitated using an inductive approach. Burnard's method was applied inductively to establish categories and abstraction through open coding illustrated in Tables 1 and 2. No single method of analysis is appropriate for every type of interview data [36]. Burnard’s method focuses on a systematic approach to thematic content analysis, which can improve qualitative research objectivity and transparency [36]. As descriptive studies can investigate perceived barriers to and facilitators of adopting new behaviours [38], a more descriptive set of themes was appropriate for the study's objectives, and it is consistent with Burnard's method [36].

## Results

A total of 20 clinicians were recruited. Eight doctors and 10 nurses were working in the polyclinics. All nurses and three doctors who participated in this study were females. The demographics of the clinicians is represented in Table 3. Demographics of clinicians (*N* = 20).

**Table 3:** Demographics of clinicians (*N* = 20)

Categories	Frequency	
Ethnicity	n	(%)
Chinese	15	(75)
Malay	2	(10)
Indian	3	(15)
Age group (years)		
25–30	3	(15)
31–35	8	(40)
36–40	5	(25)
41–45	2	(10)
46–50	2	(10)
Health professional qualifications		
Bachelor of Medicine and Bachelor of Surgery	10	(100)
Master of Medicine in Family Medicine	7	(70)
Fellowship programme, College of Family Physician Singapore	2	(20)
Graduate Diploma in Family Medicine/Physician	5	(50)
National ITE Certificate in Nursing	1	(10)
Diploma in Nursing	5	(50)
Bachelor in Nursing	9	(90)
Advanced Diploma in Nursing	2	(20)
Specialised Diploma in diabetes management and education	2	(20)

### Thematic analysis

**Fig. 1:**
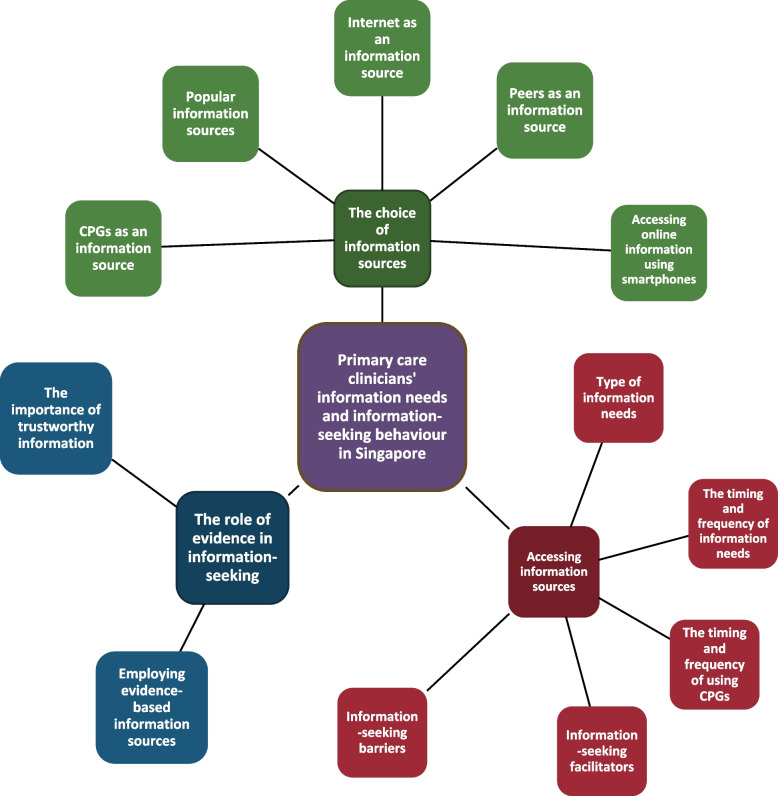
Themes and subthemes derived from the interviews

Three distinct themes were derived from the analysis of the interview data, 1) the choice of information sources, 2) accessing information sources, and 3) the role of evidence in information-seeking [see Additional file 3]. This is represented in Fig. 1. Themes and subthemes derived from the interviews.

#### 1) The choice of information sources

Is a theme that encompasses different sources clinicians in our study used to seek and gather information. Clinicians' preferred choice of information sources in five subthemes: popular information sources, CPGs as an information source, internet as an information source, peers as an information source and accessing online information using smartphones

##### Popular information sources

Clinicians mentioned that their first choice point-of-care evidence-based online sources were UpToDate®, an evidence-based resource that helps clinicians make decisions and informs their practice [39], CPGs and the Monthly Index of Medical Specialties, followed by PubMed (Medline) and continuing medication education sources. A non-evidence-based information source, the Google search engine was commonly mentioned as well. Lastly, clinicians often mentioned consulting their colleagues:

“I will Google, look for images and compare…I tell them that I’m looking because I am not sure, and I want to just confirm…sometimes even show them the photo on my phone, to ensure…what they saw, the rash…might have already disappeared is…what I suspect it is.” Doctor02.

“I commonly I would search…this app that I have on my phone is called UpToDate®, right…because it’s the most easiest…easily accessible source of information…I’ll just type the whole lot into…the Lexicomp component of the UpToDate® and then from there it tells me whether the drugs have interactions, what kind of interactions.” Doctor07.

##### CPGs as an information source

 Clinicians mentioned that CPGs did not apply to all patients. Doctors described CPGs as evidence-based resources, designed to be safe and most relevant to practice as a baseline reference. Doctors considered CPGs lengthy at times and there was a need to apply clinical discretion when using them. Doctors also mentioned that CPGs focused sometimes on cost-effectiveness instead of the quality of care:

“I think they are useful in summarising the latest evidence and what…is recommended, especially if they are local clinical practice guidelines, then it’s tailored to our own population…And keeping in mind perhaps the cost sensitivities, cost effectiveness” Doctor02.

Nurses said that they saw CPGs as a standard of practice for clinicians and an easy resource to refer to. However, some nurses said that they found CPGs difficult to access and outdated:

“…but it’s not so…easy to access…because you have to…enter certain keywords, and sometimes it’s not that keyword that’s going to churn out all the information you see…like, try a few times…want to make sure that…I’m doing things correctly…following the guidelines…just quickly…log into the intranet and…search for the information.” Nurse01.

If nurses had difficulty accessing CPGs, they said that they tended to seek doctors’ opinion:

“It’s very informative. It’s quite clear, easy to refer to…in certain special cases…not stated in the book, we will still have to seek…doctor’s opinion” Nurse07.

##### Internet as an information source

Clinicians mentioned that the internet provided access to clinical information for practice. However, clinicians mentioned that it was important to ensure that the information was well-grounded and dependable:

“…some…information might not be…so trustworthy…takes…a little additional filtering process before…I can say this is a reliable source or not…some of the websites…more opinion-based…very high…chance of bias…the reference from that writing…written at the bottom where I can do…cross-checking…I think the credibility…for this…article written is slightly higher.” Doctor01.

“If only you have an internet, you can always show it to the patient also. For example, when I search for some information, I can even help in patient education…for now, I feel it is a bit harder…And then I have to rely on my phone to use the UpToDate®.” Doctor03.

##### Peers as an information source

Clinicians mentioned approaching peers who were available to seek a second opinion on their clinical questions. They also mentioned that they tended to approach experts:

“…it’s really a case-to-case basis and it depends if the colleagues around…Also it depends on the proximity of the colleague. If the colleague knows a lot but…busy in another room on another level then I might approach next door colleagues instead.” Doctor06.

“I think most of time, if we are going to get our information immediately, we’ll call one of our colleagues here…discuss the case…we’ll come to a consensus, what will be the best for our kind of patient…contribute to the informed decision immediately.” Doctor01.

##### Accessing online information using smartphones

Clinicians mentioned that their smartphones were convenient for accessing information for practice. For instance, accessing the UpToDate® app and Google search engine using smartphones:

“…commonly I would search…this app that I have on my phone is called UpToDate®…because it’s the most easiest…easily accessible source of information…I’ll just type the whole lot into…the Lexicomp component of…UpToDate® and then from there it tells me whether the drugs have interactions.” Doctor07.

“I will go on the internet…if I needed information about…certain medical conditions…Just definitions, just to have an idea of, you know… Correct, pure Google.” Nurse01.

#### 2) Accessing information sources

Is a theme that encompasses different aspects of information-seeking and access by clinicians in our study. Factors influencing clinicians’ utilisation of information sources in five subthemes: type of information needs, the timing and frequency of information needs, the timing and frequency of using CPGs, information-seeking facilitators and information-seeking barriers.

##### The type of information needs

Clinicians mentioned that they commonly sought information on less common health areas such as unusual skin rashes, rare diseases, paediatrics, women’s health, medications, and at times concerning all clinical areas:

“Drug information…maybe dosing and everything…when we are prescribing for paediatric…we also see female patients who are pregnant…Lactating, and all… contraindicated” Doctor03.

“Other ones that I would search for would be if the patient comes in with very…unusual presentations.” Doctor07.

##### The timing and frequency of information needs

Clinicians explained that they commonly seek clinical information daily or several times a week. They said that they either seek information at the point-of-care or at home:

“I will look at least weekly once…It’s of my own interest…Not during working times, most of the time…When we are travelling, in MRT…Sometimes at home also.” Nurse10.

“Not so many cases…It’s quite rare, actually…Because most of our cases are quite common…we still can deal with…Yes…Maybe once a few weeks…Once a month…When I have concerns or any doubts…After patient left…yes. Maybe, sometimes…And after the doctors consult.” Nurse05.

##### The timing and frequency of using CPGs

Clinicians said that they commonly use CPGs daily or when there was a change or update to the CPGs:

“…day to day, because all these guidelines I’m familiar with, it’s in my memory…internally we do have guidelines for certain acute conditions.” Doctor02.

“Not so many cases…It’s quite rare, actually…Because most of our cases are quite common…we still can deal with…Yes…Maybe once a few weeks…Once a month…When I have concerns or any doubts…After patient left…yes. Maybe, sometimes…And after the doctors consult.” Nurse05.

Clinicians discussed convenience, easy access, the trustworthiness of information, having colleagues who are specialists, and being keen to keep up-to-date as the facilitators to seeking clinical information:

#####  Information-seeking facilitators

Clinicians discussed convenience, easy access, the trustworthiness of information, having colleagues who are specialists, and being keen to keep up-to-date as the facilitators to seeking clinical information:

“I find…clinical practice guidelines quite useful…since it’s on our terminal. I do open that up to look at it…it does give us quite a convenient and no fuss way to be able to access them on our terminal while we are seeking information whether during or even after consults.” Doctor06.

“work instructions…Policies and protocols…Intranet…So I just want to make sure that…I’m doing things correctly, that I’m, you know, following the guidelines. So I’ll just quickly enter, you know, log into the intranet and just search for the information…The information that’s on the intranet has, you know, been validated by an expert, you know…So that’s why I rely heavily on it.” Nurse01.

##### Information-seeking barriers

Clinicians mentioned that internet surfing separation, the lack of time, limited access to medical literature databases, and limited search function in the organisation’s server were barriers to seeking clinical information:

“The information I know is there…But it’s not so easy to search for…Not user-friendly, not very exhaustive…Sometimes you just have to…trial-and-error…different keywords.” Nurse01.

Additionally, clinicians frequently mentioned using smartphones to access clinical information. Consequently, doctors said that they were worried that using smartphones during a clinical consultation might make them seem unprofessional to patients:

“I need to explain to the patient that…I am using my phone because I don’t have internet access or may appear rude to the patient; I am surfing my phone in the middle of the consult.” Doctor02.

Doctors reported that they were also concerned about their privacy when they showed their smartphones to their patients:

“…sometimes…you don’t want to show your phone to them(patients) also…Because sometimes you may have other notifications.” Doctor05.

#### 3) The role of evidence in information-seeking

Is a theme that explores the role of evidence in clinicians' information-seeking in our study. The value of scientific research for clinicians seeking information in two subthemes: the importance of trustworthy information sources and employing evidence-based information sources.

##### The importance of trustworthy information sources

Clinicians agreed that peer-reviewed clinical information was reliable. Additionally, doctors expressed trust in clinical information if there were frequent updates of the content:

“…they(UpToDate®) do put…the date of which they have updated the articles…it’s from multiple sources…citations and…management…seems quite sound.” Doctor06.

“The information that’s on the intranet has…been validated by an expert.” Nurse01.

##### Employing evidence-based information sources

Clinicians mentioned that emphasising the importance of evidence in patient care and building an evidence-based culture in the workplace helps to encourage the use of evidence-based information sources in practice.

“I don’t have any concrete kind of suggestions now but…perhaps find some ways to sustain interest…to remind us that we’re doing this for best of patients.” Doctor06.

“If I have discussions with my peers regarding cases then I will, like, refer back to the…to…the CPG and things like that…I think the conference…or the…forums they are also a very good source of information.” Nurse03.

## Discussion

To our knowledge, this is the first study conducted in Singapore to investigate the primary care clinicians’ preferences for, barriers to, and facilitators of information-seeking in clinical practice. Clinicians’ mostly researched information on conditions such as unusual skin rashes, rare diseases, paediatrics, and women's health. Most clinicians searched clinical information at the point-of-care daily for a variety of reasons, including personal interest, clarification of doubts, or self-improvement. Sources of information included CPGs, online evidence-based resources, the internet, peers, and smartphones. Although CPGs were clinicians' preferred sources of information, they did not refer to them regularly and only did so in memory or when the guidelines were updated. We also found that using smartphones for seeking clinical information was commonly reported among clinicians. The barriers to primary care clinicians’ information-seeking process were the lack of time, internet surfing separation of work computers, limited search function of their organisation’s server, and limited access to medical literature databases. The facilitators to primary care clinicians’ information-seeking process were convenience, ease of access, and the trustworthiness of the information sources.

Like other studies [3, 8, 20, 40, 41], we found that the choice of information sources was affected by the trustworthiness and availability of resources. CPGs were preferred among clinicians as they were written by experts or specialists in their field. However, some clinicians felt that CPGs were too lengthy to be used at the point-of-care, outdated, and difficult to locate on their organisation's server. Additionally, clinicians only referred to CPGs recalled from memory or when they were updated. This highlights the importance of providing an alternative evidence-based clinical resource that is succinct and easy to refer to at the point-of-care [42]. Using medical apps for the provision of point-of-care summaries may mitigate the challenges of using CPGs for clinical information. Correspondingly, clinicians in the polyclinics commonly referred to the UpToDate® app provided by their organisation as a point-of-care resource they could use on their smartphones. Evidence-based point-of-care resources are commonly presented in key point summaries, follow formal categorisation of medical conditions, and provide references [43]. Limited research has shown that it was beneficial to integrate UpToDate® searches into daily clinical practice [42]. Additionally, the American Accreditation Commission International's @TRUST programme is one framework designed to encourage trustworthy online content. It is an invaluable resource for both individuals looking for health information online and organisations attempting to deliver trustworthy content [44]. However, continual efforts are required to encourage its use and ensure that individuals have access to accurate and reliable health information online. Therefore, future studies should investigate the quality of existing medical apps in providing point-of-care summaries and the effects of their use in the primary care setting.

We also found that clinicians were seeking clinical information on their smartphones. This is not surprising as Singapore’s public healthcare institutions enforce internet surfing separation on work computers. Furthermore, with the high penetration of smartphones in Singapore [45], these devices became the next best alternative for clinicians to seek online clinical information. Clinicians in the polyclinics frequently cited using UpToDate® app and the Google search engine on their smartphones. Similar to another study [46], we found that doctors often used Google images on their smartphones to identify less common rashes. Additionally, our study found that clinicians use Google images to educate patients. However, clinicians in the polyclinic reported privacy and professionalism concerns as barriers to using smartphones for clinical consultations. These findings were consistent with a systematic review assessing the challenges and opportunities of using mobile devices by healthcare professionals [47]. Despite the internet surfing separation in public healthcare institutions in Singapore and the availability various information sources, we found similar barriers to clinicians seeking clinical information with other studies [3, 20, 48]. Future research may focus on addressing specific barriers to using various mobile devices by primary care clinicians at the point-of-care.

Finally, smartphones may be an important information-seeking channel for healthcare professionals, and the hospital or government may be forced to establish legislation to protect healthcare professionals who use smartphones in clinical practice. Compliance with legislation governing smartphone use at work may be examined during the evaluation process for healthcare professionals. Guidelines on smartphone use among healthcare professionals can be tailored to individual conditions, such as patients' permission to share medically sensitive information via text. As a result, guidelines could be based on best practice claims and common actionable statements. Additionally, this study suggests that clinicians have, for the most part, been left to navigate information access on their responsibility, which may not be the most effective. Developing a more robust culture of evidence-based medicine within the organisation is essential and ought to be explicitly promoted moving forward. It could be beneficial for clinicians to receive organised training on effective information-seeking strategies and resources.

Our study has several strengths and limitations. Our strength is that we employed an in-depth interview approach and an open-ended style of questioning. The interactive nature of our interviews provided richer context and room for free responses from the interviewees. We were then able to critically scrutinise the conversations and provide insights that were helpful in the final analysis of themes.

There are several limitations. Firstly, we did not explore the influence of gender and age in the participants’ information-seeking behaviour, which has been demonstrated in other research in this area [14]. Secondly, the study was limited by environmental factors in the workplace, such as internet and information access. Finally, there may be possible social desirability bias, whereby the participants may have presented responses that were more socially appropriate than their actual thoughts on the issues explored during the interviews.

## Conclusion

We found that clinicians frequently sought answers to clinical queries arising from patient care. However, the choice of information sources was influenced by the trustworthiness and availability of the resources. Clinicians in the polyclinic commonly reported using their smartphones for practice. Using UpToDate® app and Google search engine was commonly cited as their preferred clinical information sources due to its convenience and accessibility. While our findings may have been reported in other contexts, there are significant and novel elements when compared to healthcare around the world. For example, the implementation of internet surfing separation in public healthcare institutions raises concerns regarding clinicians' usage of smartphones, as well as their privacy and professionalism. This may lead us to examine the need for some regulation and training on the use of smartphones among clinicians, as well as the necessity to investigate this further from the patient's perspective. Future studies to improve access to evidence-based clinical information sources other than CPGs should be explored to address the information needs of primary care clinicians. Studies examining trustworthiness and effectiveness of using app-based point-of-care information summaries and exploring the impact of using mobile devices for information-seeking by clinicians at the point-of-care will also be useful to address the information-seeking needs of primary care clinicians. Furthermore, Large Language Model (LLM)-based artificial intelligence (AI) systems, such as ChatGPT, are increasingly being developed and used. They are used in various disciplines, including healthcare. Some, such as AMIE (Articulate Medical Intelligence Explorer) and Pathways Language Model (Med-PaLM 2), have been developed specifically for healthcare [49–51]. More research into the usage of AI among clinicians is needed to assure trust, dependability, and ethical conduct.

### Supplementary Information

Supplementary Material 1.

Supplementary Material 2.

Supplementary Material 3.

Supplementary Material 4.

## Data Availability

The datasets generated and/or analysed during the current study are not publicly available due the fact that all data obtained during the course of this study is strictly confidential and will be kept by the study team at the end of the study for at least 6 years and disposed of according to the Personal Data Protection Act in Singapore. Data are however available from Associate Professor Tang Wern Ee (co-author) upon reasonable request and with permission of the ethics committee of National Healthcare Group Domain Specific Review Board (the central ethics committee).

## Abbreviations

GP: General Practitioner
CPG: Clinical practice guidelines
