# Supplementary material for: Identifying primary care clinicians’ preferences for, barriers to, and facilitators of information-seeking in clinical practice in Singapore: a qualitative study
Source: BMC Prim Care. 2024 May 18;25:172. doi: 10.1186/s12875-024-02429-x (PMC11102200; doi:10.1186/s12875-024-02429-x)
Supplement: Supplementary file 2 — Supplementary Material 2. [file 12875_2024_2429_MOESM2_ESM.docx]

| **Subject ID:** | IDI | DR or NR |  |  |  |  |
| --- | --- | --- | --- | --- | --- | --- |


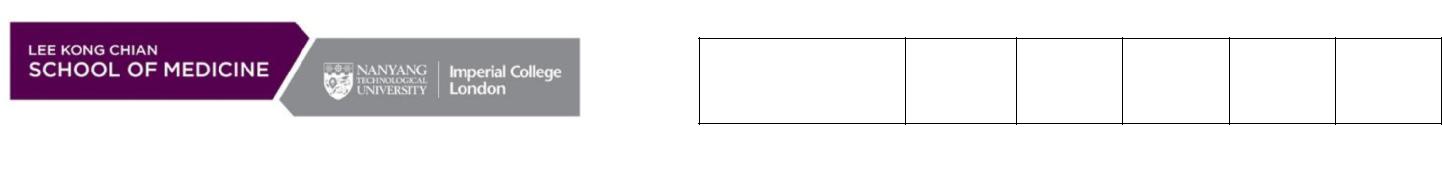
 In-depth interview Doctor or nurse Interviewer Initials Sequential number

Protocol Title:

**Information seeking behaviour of primary care practitioners in Singapore:
finding evidence to support high-quality patient care**

In-depth Interview Topic Guide

**Introduction**

We are interested in exploring the information sources, preferences as well as barrier and facilitators to information seeking. We would like to invite you to participate in an approximately 60 minutes one-to-one semi-structured interview, to share your views on motivations and challenges to information seeking. The interview will be recorded but pseudonyms are used for the purpose of transcription, analysis and publication. All information obtained in this interview is strictly confidential and will be kept securely by the study team at the end of the study for 6 years and disposed of according to the Personal Data Protection Act.

**Information needs**

1. What type of information do you search for the most in clinical practice?
2. Which clinical areas do you find yourself facing the most questions in?

**Information sources**

1. Where do you usually get patient care-related information? Why?
2. How often and when (in the morning/afternoon/evening) do you search for information?
3. What is your view on clinical practice guidelines? Do you commonly use clinical guidelines in your practice? If no, why not? If yes, how often do you estimate you use the guidelines (daily, few times a week etc.)?
4. What is your view on internet sources/webpages to as a source of information for clinical practice? Do you commonly use internet sources in this way? If yes, can you please name the sources used?
5. What is you view on seeking advice from peers on clinical practice?
6. Which source of information do you trust the most? Why?

**Preferences and barriers to information in clinical practice**

1. What are the main barriers that prevent you from searching for information? (eg. time, not sure how to use databases, unclear where to search or how)
2. How satisfied are you with current information sources? Do you feel they are lacking in any ways/areas? If so, which areas and how do you think they can be improved?
3. What suggestions do you have for encouraging health professionals like yourself to utilise different types of evidence-based resources?

We have come to the end of the interview. Is there anything not covered but you would like to add? If not, kindly complete the questionnaire provided.

| Interviewee ID: | DR | - |  |  |  |
| --- | --- | --- | --- | --- | --- |

**Please complete the section below:**

**Age: 🞎** 25 – 30 years old Ethnicity: **🞎** Chinese
 **🞎** 31 – 35 years old **🞎** Malay
 **🞎** 36 – 40 years old **🞎** Indian
 **🞎** 41 – 45 years old **🞎** Others (Please specify):
 **🞎** 46 – 50 years old ___________________
 **🞎** 51 – 55 years old
 **🞎** 56 – 60 years old
 **🞎** > 60 years old

**Health Professional Qualification**

| **Year Obtained** | **Qualification (Major)** (Bachelor, Masters, PhD) | **Institution & Country** |
| --- | --- | --- |
|  |  |  |
|  |  |  |
|  |  |  |

**Thank you for your time and effort in participating in this interview.**
