# Supplementary material for: Identifying primary care clinicians’ preferences for, barriers to, and facilitators of information-seeking in clinical practice in Singapore: a qualitative study
Source: BMC Prim Care. 2024 May 18;25:172. doi: 10.1186/s12875-024-02429-x (PMC11102200; doi:10.1186/s12875-024-02429-x)
Supplement: Supplementary file 3 — Supplementary Material 3. [file 12875_2024_2429_MOESM3_ESM.docx]

**Themes and subthemes**

| **The choice of information sources** | **Accessing information sources** | **The role of evidence in information-seeking** |
| --- | --- | --- |
| Popular information sources | Type of information needs | The importance of trustworthy information sources |
| CPGs as an information source | The timing and frequency of information needs | Employing evidence-based information sources |
| Internet as an information source | The timing and frequency of using CPGs |  |
| Peers as an information source | Information-seeking facilitators |  |
| Accessing online information using smartphones | Information-seeking barriers |  |

**Themes, subthemes and codes**

| **The choice of information sources** | | |
| --- | --- | --- |
| **Popular information sources** | | |
| **Evidence-based resources** | | |
| **Codes** | **Definition (inclusion – keywords – and exclusion criteria)** | **Quotes** |
| ***First choice point-of-care evidence-based online sources***   - UpToDate (Common among doctors and nurses) - CPGs (Common among doctors and nurses) - MIMS (Common among doctors and nurses) - DermNet NZ (Common among doctors) - SANFORD (Common among doctors) - Medscape (Common among doctors) | The preferred evidence-based online sources used among participants to seek answers to their clinical questions. | “I commonly I would search…this app that I have on my phone is called UpToDate, right…because it’s the most easiest…easily accessible source of information…I’ll just type the whole lot into…the Lexicomp component of the UpToDate and then from there it tells me whether the drugs have interactions, what kind of interactions.” Doctor07  “I find that (CPGs) quite useful as well because…since it’s on our terminal. I do open that up to look at it, yes…it does give us quite a convenient and no fuss way to be able to access them on our terminal while we are seeking information whether during or even after consults.” Doctor06  “Medscape is fast…if I really need…my question answered very quickly, then I look for it there.” Doctor10 |
| ***Second choice evidence-based online source***   - Literature search engine: PubMed (Medline) (Common among doctors and nurses) - Continuing medical education (Common among doctors and nurses) - Medical education websites (AAFP, Mayo and Cleveland clinic) (Common among doctors) - Medical education websites (clinical web, WebMD, Mayo clinic, Medscape, Immunisation Coalition, local health ministry and U Central) (Common among nurses) - Medical journals (Singapore Medical Journal, Singapore Family Physician and BMJ best practice) (Common among doctors) - Local medical council (including articles in newsletters from local hospitals, written by new consultants) (Common among doctors) - Resources for medications (PharmD Live and drugs.com) (Common among doctors) | The next best option of online evidence-based sources should the preferred choice be unavailable or insufficient to provide answers to the participants’ clinical questions. | “sometimes I do try to use PubMed when I have some questions…That…cannot be easily found on these other sources that I mentioned…for this year I can only recall one instance…The information cannot be readily found on…UpToDate…I had to look through like individual papers in order to find whether they have an answer on that.” Doctor05  “I think their (AAFP) information’s quite clear and…it’s easy to follow…you don’t want…website where everything’s very wordy. And then you…get confused and lost when you’re trying to find information…I think their information is very clear and it’s very precise.” Doctor07  “(Singapore Medical Journal and Singapore Family Physician) those will be when really have time, then I can look through them.” Doctor10 |
| ***Second choice evidence-based printed source (Common among nurses)***  • Books written by the hospital | The evidence-based printed source is the next best option of evidence-based source should the online evidence-based choices be unavailable or insufficient to provide answers to the participants. | “so the KK, the booklet…it’s related to children development also…But we can still like, you know, (look for) certain signs and symptoms, you know…what the child will exhibit and all this will be…Similar.” Nurse10 |
| **Non-evidence-based resources** | | |
| ***Non-evidence-based online sources***   - Search engine (Google) (Common among doctors and nurses) - Online encyclopedia (Wikipedia) (Common among nurses) - Social media such as Facebook, forum and newspapers (Common among nurses) | The non-evidence-based online sources that participants use in their clinical practice. | “I will Google, look for images and compare…I tell them that I’m looking because I am not sure, and I want to just confirm. And I sometimes even show them the photo on my phone, to ensure that what they saw, the rash…that they saw, they might have already disappeared is according to what I suspect it is.” Doctor02 |
| **Colleagues (Common among doctors and nurses)** |  |  |
|  | Participants indicated their preference to turn to their co-workers for answers to their clinical questions. | “sometimes, unless we know that they have been doing that particular method or that particular treatment for a long time, and…we just want to ask them how’s the experience or how they do it… It’s more anecdotal…rarely because…things are a bit more easily available on the internet.” Doctor10  “I would then think which of my colleagues is best pitched to answer these questions, yes. So if I’m faced with a challenging issue definitely, I’ll probably ask more of a senior or my clinic head for example…Someone who I know that I will trust to know that information, yes.” Doctor06  “Sometimes, if we’re, usually…if, really, if you are not very sure, we can always ask our seniors…And the champions in the service area…Because…maybe they’re more experienced, yeah, they exposed to like more special cases or more challenge one…We always can ask them. Sometimes, I also ask doctors. Also, can ask our NM, nurse manager, yeah.” Nurse05 |
| **CPGs as an information source** | | |
| CPGs are not applicable to all patients (Common among doctors and nurses) | Participants think that CPGs are impractical to poorly defined patient population. | “each patient is individual. There will be reasons why I would need to deviate from the clinical practice guidelines…in my documentation for the notes, I will write why, because in our current medical legal climate, if you don’t follow guidelines, you deviate, you should justify why…” Doctor02 |
| CPGs are evidence-based practice (Common among doctors) | Participants believe that CPGs are summaries of peer-reviewed recommendations. | “I think they are useful in summarising the latest evidence and what…is recommended, especially if they are local clinical practice guidelines, then it’s tailored to our own population...And keeping in mind perhaps the cost sensitivities, cost effectiveness” Doctor02 |
| CPGs are designed to be safe (Common among doctors) | Participants suggested that CPGs are drawn up with the intention to guard patients and practitioners. | “The CPGs are designed to be safe, if you follow them…you are unlikely to…harm the patient…you have understood the…CPG…and you have very good reasons to divert…from it…defensible.” Doctor04 |
| Some CPGs are relevant to practice (Common among doctors) | Participants suggested that some CPGs are applicable to patients with single morbidity without complications. | “I believe now with the…more bite-size appropriate care guideline, where they are talking about single…disease, single conditions…sometime even on a single…issues…more relevant to practice itself” Doctor01 |
| Some CPGs are lengthy (Common among doctors) | Participants suggested that some CPGs are too wordy and are not easy to use for practice. | “Used to have a comment for our local Singapore CPG, where there are a lot of evidence-based-related thoughts inside, and very lengthy descriptions about why certain things has to be done like this. Without take into consideration, how…the actual management workflow is like…there’s a lack in…how are we going to implement and manage accordingly…, as in how this thing’s being translate into practices.” Doctor01 |
| There is a need to apply clinical discretion when using CPGs (Common among doctors) | Participants believe that clinical judgment is required when adopting CPGs. | “knowing that we are deriving that information from…evidence-based medicine, where…inherent problems related to evidence-based medicine…there are also limitations in application to your…patient…to be fair, the clinical practice guidelines do state that you apply clinical discretion…you can decide if this is my, is applicable to your patient.” Doctor04 |
| CPGs may focus on cost effectiveness and not quality of care (Common among doctors) | Participants believe that CPGs may be centred on providing cost-efficient services than the health outcomes for patients. | “where MOH is heading now is not just clinical practice guidelines, but they have agency for care effectiveness. So, they are integrating clinical practice guidelines with cost effective care. So, I am not sure whether that’s a good thing or a bad thing because they may not be pushing what the evidence shows to the best care, but what is most cost effective for the government.” Doctor02 |
| CPGs as baseline reference that is applicable to most patients in the local population. (Common among doctors) | Participants suggested that some CPGs are standard recommendation applicable to most local patients. | “most times, I will try, as much as possible, to follow (Ministry) clinical Guidelines if they are available…(they) are very local-based” Doctor10 |
| CPGs provide standard practice for clinicians (Common among nurses) | Participants suggested that some CPGs are standard recommendations for practice. | “It’s there for a reason, good for standardization purpose, make sure everyone does the same…thing correctly.” Nurse01 |
| CPGs may not be updated regularly (Common among nurses) | Participants mentioned that CPGs are not frequently revised and may not be providing up-to-date advise. | “sometimes, you do realise that the information is not updated…I would…say maybe about once a month, I would receive an email to say that, oh, there is a new version of this thing…but the thing is…it’s not like they revise everything…certain things…If it’s related to you, then you would want to read it. If it’s not, then you just…ignore it…if it’s not updated, there must be a reason.” Nurse01 |
| CPGs are not easy to access (Common among nurses) | Participants mentioned that CPGs are not easily obtainable when navigating in the organisation’s database. | “but it’s not so…easy to access, you…because you have to…enter certain keywords, and sometimes it’s not that keyword that’s going to churn out all the information you see…kind of like, try a few times…So I just want to make sure that…I’m doing things correctly, that I’m, you know, following the guidelines. So I’ll just quickly enter, you know, log into the intranet and just search for the information.” Nurse01 |
| CPGs are easy to refer (Common among nurses) | Participants suggested that CPGs are simple to access and understand. | “It’s very informative. It’s quite clear, easy to refer to. But of course, in certain special cases that’s not stated in the book, we will still have to seek a doctor’s opinion” Nurse07 |
| **Internet as an information source** | | |
| Importance of credibility (Common among doctors and nurses) | Participants are prudent in the use of online clinical information. They ensure that the information is well-grounded and dependable. | “Where some of the information might not be…so trustworthy… takes…a little additional filtering process before…I can say this is a reliable source or not… some of the websites, they are mainly more opinion-based…there is actually very high…chance of bias…the reference from that writing itself is written at the bottom where I can do a kind of cross-checking…I think the credibility…for this…article written is slightly higher.” Doctor01 |
| Internet provides access to clinical information for practice (Common among doctors and nurses) | Internet is perceived as a convenient and easy to access source. | “if only you have an internet, you can always show it to the patient also. For example, when I search for some information, I can even help in patient education…for now, I feel it is a bit harder…And then I have to rely on my phone to use the UpToDate.” Doctor03 |
| Years of experience as a clinician impacts search for clinical information (Common among doctors) | The vast experience obtained by the participants influences how the participants use internet as an information source. | “because of the cases that I see…and the experience that I gain for the past few years…less likely for me, there is a need for me to do additional searches…but then if you are talking about looking to new area...For research or for education…Creating education material, sometime we will do…literature search, to ensure that we are not missing out new updates…I will do this every day.” Doctor01 |
| Tend not to use internet sources for clinical information (Common among doctors) | Participants mentioned that they do not using internet sources for clinical information. | “the information is too…heterogeneous…even if…is from a fairly reputable source like Medscape…the practice…may not be the same…they may be applying an alternative guidelines.” Doctor04 |
| Dependent on which level of evidence one is keen to acquire (Common among nurses) | A well-established internet source is required to retrieve high-level evidence information for practice. | “Depends on which level you want to be at…If you want to put in practice, of course the…RCT I know, but if you're just for knowledge, sometimes expert also can…so it depends on which level you want to get…and whether it’s too chiem (hard) to understand or not. Some level I don't understand…So depends on…If it's just leisure reading…sometimes I just Google like this and see what… But if I really want good, at least the scholar” Nurse02 |
| **Peers as an information source** | | |
| Tapping the expert (Common among doctors and nurses) | Participants would look for specialists to seek answers to their clinical questions. | “If I know, for example, in our clinic we have a dermatology champion, so they have already completed their graduate diploma in dermatology…Then I will ask them for help, for particular dermatological condition.” Doctor02 |
| Seeking second opinion from peers (Common among doctors and nurses) | Participants speak to their colleagues to gain a different perspective on issues. | “we can have, sometimes, but not…as a routine, you know…not the usual practice, but when we are in doubt, we used to refer to the guidelines (“Rather than we go out and ask people”) If we still need any further clarification, we used to ask our peers…we can also get help from them.” Nurse10 |
| Availability of colleagues affect seeking advice from peers (Common among doctors and nurses) | Participants are unable to approach their peers for clinical answers if they are busy or unavailable. | “it’s really a case-to-case basis and it depends if the colleagues around…Also it depends on the proximity of the colleague. If the colleague knows a lot but he’s busy in another room on another level then I might approach next door colleagues instead for example, yes.” Doctor06 |
| Seeking common consensus from peers (Common among doctors) | Participants speak to their colleagues to gain support on issues. | “I think most of time, if we are going to get our information immediately, we’ll call one of our colleagues here…discuss the case…we’ll come to a consensus, what will be the best for our kind of patient…contribute to the informed decision immediately.” Doctor01 |
| Seeking advice from peers for non-medical information (Common among doctors) | Participants speak to their colleagues for non-clinical information. | “may not be medical questions…How do you, uh, handle MOH audits?...How’s the CHAS doing” Doctor10 |
| Perceived that colleague is not keen to assist or appreciate advice given (Common among nurses) | Participants mentioned that co-workers appear aloof when approached for information. | “seeking advices from the peers I think…it’s quite good…because we…wants to know their opinions. But however sometimes when you seek advice also they…actually not keen to tell you when they are occupied…Can be my fellow nurses or fellow doctors…And sometimes also when you give advice, some advices, uh sometimes they might feel agitated…I usually ask…let’s say if I got no choice, if I’m seeing a patient, I will just have to, sorry…give me a moment.” Nurse04 |
| **Accessing online information using smartphones** | | |
| Using personal smartphones to access UpToDate (Common among doctors and nurses) | Participants mentioned using their personal smartphones for seeking information for practice. | “I commonly I would search…this app that I have on my phone is called UpToDate, right…because it’s the most easiest…easily accessible source of information…I’ll just type the whole lot into…the Lexicomp component of the UpToDate and then from there it tells me whether the drugs have interactions, what kind of interactions.” Doctor07 |
| Using personal smartphones to access Google search engine (Common among doctors and nurses) |  | “I will go on the internet…if I needed information about…certain medical conditions…Just definitions, just to have an idea of, you know… Correct, pure Google” Nurse01 |
| **Accessing information sources** | | |
| **The type of information needs** | | |
| **Codes** | **Definition (inclusion – keywords – and exclusion criteria)** | **Quotes** |
| Information on less common conditions (Common among doctors and nurses) | Participants search for information on conditions that are not commonly encountered in their practice. | “Other ones that I would search for would be if the patient comes in with very…unusual presentations.” Doctor07 |
| Paediatrics and Women’s health  (Common among doctors and nurses) | Areas that involve the treatment of women’s and children’s health conditions. | “if I were to have an area… where…I might need to clarify…that… would probably be the…Paediatrics, maybe…Gynecology…presenting a bit atypically.” Doctor04 |
| Drug-related information for treatment  (Common among doctors and nurses) | Participants search for drug-related information (i.e., name, side-effects and contraindications of the drug) including and not limited to pharmacological and pharmacy details. | “Drug information…then maybe dosing and everything…when we are prescribing for paediatric…Patients… we also see female patients who are pregnant…Lactating, and all... contraindicated” Doctor03 |
| All clinical areas  (Common among doctors and nurses) | Participants provided general description of having questions in several clinical areas. | “it’s very, very broad because…family practice, right, it’s just very, very broad…So, sometimes you just want to be really safe, you know…or even I check it almost every day” Doctor07 |
| Diagnosis-related information  (Common among doctors) | Participants search for medical information and current best practice to derive a diagnosis, to resolve a diagnostic dilemma and/or to help in managing patients’ conditions. | “we look for medical information when we have difficulty in diagnosing certain conditions, or we have to look out for certain…common symptoms, or we need to find out some diagnosis…we need to…find more information with regards to any medical condition.” Doctor03 |
| Information for patient education  (Common among nurses) | Participants search for advice to provide to patients. | “for example when I deal with the children…For example…make sure the behaviour, some parents will ask us whether the behaviour is normal or not…Then make sure I check whether the milestones is correct for the particular age… if for example, the patient ask any other questions. Like, for example, they want to know much more information…If they still they don’t know, then I will look for the information also… Mostly some patient…about the education part…they will ask also. Like, for example, particular whether they can take this one or not.” Nurse08 |
| Information in the acute setting (Common among nurses) | A healthcare setting that focuses on triaging, dealing with unplanned cases or procedures. | “the acute care setting, because the patients…they are not, like…appointment-based, very random cases.” Nurse01 |
| **The timing and frequency of information needs** | | |
| **Frequency** | | |
| Daily (Common among doctors and nurses) | An indication that participants seek information for practice every day. | “once or twice per day maybe…When we really need to prescribe.” Doctor10 |
| Several times a week (Common among doctors and nurses) | An indication that participants seek information for practice couple times per week. | “CPG…very frequent, probably, like, twice a week” Doctor07 |
| Once a month (Common among nurses) | An indication that participants seek information for practice monthly. | “Once a month…not frequent. (to seek information on Google)” Nurse06 |
| **Timing** | | |
| At the point of care (Common among doctors and nurses) | An indication that participants seek information at patient’s bedside. | “maybe about four times a week…Quite frequent…At the point of care…At my service station.” Nurse01 |
| At home (Common among doctors and nurses) | An indication that participants seek information when they are at their own residence. | “it can be any time of the day…And if it’s not as urgent then you can do it at home.” Doctor06 |
| After consultation (Common among nurses) | An indication that participants seek information following patient’s medical check. | “Not so many cases…It’s quite rare, actually…Because most of our cases are quite common…we still can deal with…Yes…Maybe once a few weeks…Once a month…When I have concerns or any doubts…After patient left…yes. Maybe, sometimes…And after the doctors consult.” Nurse05 |
| When commuting (Common among nurses) | An indication that participants seek information when they are shuttling between places. | “I will look at least weekly once…It’s of my own interest…Not during working times, most of the time…When we are travelling, in MRT...Sometimes at home also.” Nurse10 |

| **The timing and frequency of using CPGs** | | |
| --- | --- | --- |
| **Frequency** | | |
| Daily (Common among doctors and nurses) | An indication that participants use CPGs for practice every day. | “day to day, because all these guidelines I’m familiar with, it’s in my memory. So, I will use it every day…Because I see chronic disease patients on a daily basis…But internally we do have guidelines for certain acute conditions.” Doctor02 |
| Twice a week (Common among doctors) | An indication that participants use CPGs for practice two times per week. | “CPG…very frequent, probably…twice a week” Doctor07 |
| Several times a month (Common among nurses) | An indication that participants use CPGs for practice couple of times per month. | “actually not so much. Because I don’t find it too…much information I can get from there…At least…it depends. If I’m doing teaching…I will do about once a week…Let’s say if I’m guiding a, a preceptee, I will have to show them the guidelines.” Nurse04 |
| **Timing** | | |
| When there is a change or update to the CPGs  (Common among doctors and nurses) | Participants refer to the CPGs when there is an amendment or development made in the guidelines. | “I will read through when let’s say there’s a…revised version…Then if I remember already, then I don’t have to refer back. It’s not a daily basis. But more of like when there’s a revised version, I will revise accordingly, or there is…something I need to clarify, then I’ll go back to it.” Nurse06 |
| When I forget something in the CPGs (Common among doctors) | Participants refer to the CPGs when they are unable to recall the details in it. | “I am longer in the Clinical…after practicing a bit more, I don’t use it that often anymore, unless…I forgot, then I will search for it.” Doctor08 |
| Use when colleagues are unavailable (Common among nurses) | Participants refer to the CPGs if their co-workers are unreachable. | “my fellow colleagues are busy with the patients…I will follow the guidelines.” Nurse04 |
| Whenever there is a rotation of service station (Common among nurses) | Participants refer to the CPGs when there is a change in their work area. | “If, let’s say, the sister suddenly change me to a new, new environment, new service station that I so long like one, two months, I haven’t… Have not been assigned there and I will look through the guideline” Nurse05 |

| **Information-seeking facilitators**  **Derive from sub-theme: popular information sources* | | | |
| --- | --- | --- | --- |
| Convenience and easy access (Common among doctors and nurses) | Participants tend to seek information when they perceived that the source is available and obtainable. | “I find that clinical practice guidelines (CPGs) quite useful as well…since it’s on our terminal. I do open that up to look at it, yes…it does give us quite a convenient and no fuss way to be able to access them on our terminal while we are seeking information whether during or even after consults.” Doctor06 | |
| Evidence accepted by experts implies trustworthiness of information (Common among doctors and nurses) | An indication that available sources that include experts’ opinions are dependable and usable by the participants. | “work instructions…Policies and protocols…Intranet…So I just want to make sure that…I’m doing things correctly, that I’m, you know, following the guidelines. So I’ll just quickly enter, you know, log into the intranet and just search for the information…The information that’s on the intranet has, you know, been validated by an expert, you know…So that’s why I rely heavily on it.” Nurse01 | |
| Having colleagues who are specialists (Common among doctors and nurses) | An indication that the presence of experts in the field makes retrieval of clinical information dependable and obtainable by the participants. | “If it’s…related to their field like…If I'm not sure about like flow or in vaccination, because I'm not trained…these are things that I can ask my trained nurses. Because I’m under chronic disease management. But the screening I also offer my patient, so if I'm not so clear about the timing and all that, then I will ask my nurses who are trained in immunisation. That’s different, depends on what area you're looking for…it's not just all the doctors too, the nursing as well…Also dietician, cannot forget dietician. Because I do counsel about diet, but if the information is too much that the patient ask me, then I'm stuck. Then I will go back to my dietician. My patient say like that…is it true or not? So that's how we also work closely with our dietician.” Nurse02 | |
| To get an idea of new information (Common among doctors and nurses) | An indication that participants are keen to do a search of information to know some background of the topic of interest. | “I will go on the internet…if I needed information about…certain medical conditions…Just definitions, just to have an idea of, you know… Correct, pure Google” Nurse01 | |
| Resource is free or provided by organisation (Common among doctors) | Participants are keen to utilise sources that are readily available and supported by their institution. | “The other source that I commonly rely on is UpToDate, which is through my phone, so, we have institution login that’s free subscription to it.” Doctor02 | |
| To retrieve immediate and accurate answers (Common among doctors) | Participants tend to seek information when they perceived that they are able to receive prompt and precise answers. | “Most of the instant answers I get is from UpToDate.” Doctor08 | |
| **Information-seeking barriers** | | | |
| Internet separation (Common among doctors and nurses) | No internet is perceived as a barrier to information seeking. | | “I need to explain to the patient that…I am using my phone because I don’t have internet access or may appear rude to the patient; I am surfing my phone in the middle of the consult.” Doctor02 |
| Time (Common among doctors and nurses) | Time pressure is perceived as a barrier to information seeking. | | “if we have a complicated patient with multiple conditions, then I may be less inclined to look for the information unless it’s critical in decision of the management of the patient…If I actually remember, I would go further, but a lot of the time I would have forgotten by the time I reach home because of the patient load.” Doctor02 |
| Limited access to subscribed databases (Common among doctors and nurses) | Restricted connection to databases is perceived as a barrier to information seeking. | | “time consuming. Or, too slow, the system run very slow…time…every job there is time pressure” Nurse02 |
| Poor search function in intranet (Common among doctors and nurses) | Incompetent algorithm to search through organisation’s database is perceived as a barrier to information seeking. | | “The information I know is there…But it’s not so easy to search for it…Not user-friendly, not very exhaustive, I find, you know… Sometimes you just have to trial, trial-and-error, you know, different keywords.” Nurse01 |
| Motivation (Common among doctors) | A weak desire in participants to answer their clinical questions is perceived to be a barrier to information seeking. | | “we all know that the resources are available…the question is whether do I go and do it or not.” Doctor06 |
| Professionalism (Common among doctors) | Not showing a professional image is perceived to be a barrier to information seeking. | | “sometimes you want to show a patient, you don’t want to show your phone to them also…Because sometimes you may have other notifications.” Doctor05 |
| Stress (Common among doctors) | Being pressured to keep pace with work guidelines is perceived to be a barrier to information seeking. | | “the fast pace of things changing…there are so many new…research projects, and information…shared, even within…the internet forum, sometime you, even one disease, you can get confused…quoting even certain…international guideline, sometime…the US guideline and UK guideline can come out, like…five months apart…some of the authors…sit in both committee...But the actual recommendation defer quite a lot…this kind of thing…you will get confuse also.” Doctor01 |
| No access to shared drive (Common among doctors) | Not being able to share information freely with colleagues is perceived to be a barrier to information seeking. | | “we could use the USB to upload these e-books into our common shared drive, it was easier to share. But now it’s no longer possible. Again, because they stopped USB.” Doctor02 |
| Lack of evidence (Common among doctors) | The lack of peer-reviewed information for drugs is perceived as a barrier to information seeking. | | “some drug reps will come and tell you…these probiotics actually very good for immune systems…if you want to find that out, it’s very tough because…the amount of studies they do is quite little… when I try to search on something, it’s very…Super depressing…because you can never find the answer.” Doctor10 |
| Lack frequent review of change in practices by senior colleagues (Common among nurses) | The lack of updates by senior co-workers on clinical practices is perceived as a barrier to information seeking. | | “I think I prefer like, for example, like, every, like, weekly or monthly we still…we have a talk by the senior people about the new changes…otherwise…revising the current one I think to keep update.” Nurse08 |
| Unsure of how to use databases provided by the organisation (Common among nurses) | The lack of competence to navigate the company’s database is perceived as a barrier to information seeking. | | “We actually have…what’s that called… The library access then that we can actually find out what you want to look out for…Intranet, I’m not sure whether we have the direct access, but for nursing part, we have the nursing…Can’t recall already…Quite a while never use that’s why…No access.” Nurse09 |
| Uncertain if information is trustworthy (Common among nurses) | The inability to ascertain dependable information is perceived as a barrier to information seeking. | | “Another thing is that…often when we’re online we don’t know where’s the source, we don’t’ know whether it is trusted or not.” Nurse03 |
| Not updated information (Common among nurses) | The infrequent update of content depicts the poor editorial quality of information and questioned the dependability of the information. This is perceived as a barrier to information seeking. | | “sometimes, you do realise that the information is not updated…I would…say maybe about once a month, I would receive an email to say that, oh, there is a new version of this thing…but the thing is…it’s not like they revise everything…certain things…If it’s related to you, then you would want to read it. If it’s not, then you just…ignore it…if it’s not updated, there must be a reason.” Nurse01 |
| Information is not conveniently located (Common among nurses) | Poor accessibility of information is perceived as a barrier to information seeking. | | “It is not available in the room itself. Yes, so accessibility.” Nurse07 |
| Perceived information provided in guidelines, protocols and colleagues as rigid and inflexible to alter (Common among nurses) | When information gathered from workplace is deemed strict and unalterable, it is perceived as a barrier to information seeking. | | “I think some barriers like…some people they say their way…This one must be done like this way…then we cannot change the one…Sometimes the guidelines also sometime…It’s like that, means like that…cannot like changed…Same also…the protocol says like that means, like, same also.” Nurse08 |
| **The role of evidence in information-seeking** | | | |
| **The importance of trustworthy information sources** | | | |
| **Codes** | **Definition (inclusion – keywords – and exclusion criteria)** | | **Quotes** |
| Peer reviewed (Common among doctors and nurses) | Information that has been peer reviewed is more dependable than non-peer reviewed information. | | “The information that’s on the intranet has…been validated by an expert.” Nurse01 |
| Date of update stated in the source (Common among doctors) | The frequent update of content depicts the editorial quality of information and ensure that information can be dependable. | | “they (UpToDate) do put also the date of which they have updated the articles… it’s from multiple sources, there’s multiple citations and, the management…seems quite sound.” Doctor06 |
| Supported by in-depth analysis (Common among doctors) | Indication that information is substantiated by rigorous investigation. | | “it depends on the scenario…I would want to know, the why behind that…reasoning…so it becomes…about…can I trust this specific source to have incorporated…the raw data that is available.” Doctor04 |
| Originating from multiple sources (Common among doctors) | Indication that information is written based on preferably systematic reviews. | | “if you talk about all the info stored within that particular source, then it will be PubMed…because…it has everything.” Doctor10 |
| Colleagues with experience (Common among doctors) | Indication that the information is derived from co-worker with vast experience in that area. | | “Peers will be more…Because…these are people sometimes you know and… they probably have done it before…Or seen it before” Doctor09 |
| **Employing evidence-based information sources** | | | |
| To emphasize the importance of evidence in patient care (Common among doctors and nurses) | An indication that peer-reviewed information should be prioritise and use when providing patient care. | | “I don’t have any concrete kind of suggestions now but…some ways to perhaps find some ways to sustain interest or…impact this and to remind us that we’re doing this for best of patients.” Doctor06 |
| To build evidence-based culture (Common among doctors and nurses) | An indication that the organisation should encourage participants to inculcate evidence-informed practices. | | “if I have discussions with my peers regarding cases then I will, like, refer back to the...to…the CPG and things like that…I think the conference…or the…forums they are also a very good source of information.” Nurse03 |
