# Supplementary material for: Identifying primary care clinicians’ preferences for, barriers to, and facilitators of information-seeking in clinical practice in Singapore: a qualitative study
Source: BMC Prim Care. 2024 May 18;25:172. doi: 10.1186/s12875-024-02429-x (PMC11102200; doi:10.1186/s12875-024-02429-x)
Supplement: Supplementary file 4 — Supplementary Material 4. [file 12875_2024_2429_MOESM4_ESM.docx]

Burnard’s method uses fourteen stages to categorise and code the interview transcripts (39). During the first stage, notes are taken after each interview to discuss various topics. In the second stage, the focus is on reading and noting general themes found in the transcripts so one can completely immerse themselves in the data. The third stage involves reviewing transcripts and creating headings to summarise each component of the content (Table 1).

Stage four involves combining similar categories into broader ones to reduce the number of categories. For example, various information sources that were mentioned by the participants such as evidence-based resources, non-evidence-based resources, and colleagues have all been merged into a single category of subthemes titled "popular information sources". In stage five, the final list is created by removing repeated or similar heads from the new category list and subheadings. In stage six, two additional study team members independently create a list of categories without exposing the first study team member's list. A discussion and improvement of three lists is conducted to improve their validity and reduce researcher bias. In stage seven, transcripts are reviewed and categories and subheadings are confirmed to make sure that every element of the interviews are addressed. At stage eight, each transcript is checked against the categories and subheadings, then 'coded' accordingly. Coloured highlighters can be used to distinguish between categories and subheadings. For example, a yellow highlight indicates online resources, a green highlight indicates patients as information sources, and a pink highlight indicates colleagues as information sources.

In stage nine, every component of all codes is compiled. All codes are compiled in stage ten under the relevant headings and subheadings. The eleventh stage involves selected respondents evaluating whether the sections are appropriate (i.e., headings, sub-headings, categories, and codes). In stage twelve, the sections are arranged for ease of access. In stage thirteen, after all sections are collated, the writing-up process begins. In the fourteenth stage, the analysis can be written by the study team member, who will use actual interview samples to illustrate each part. Next, think about contrasting such analyses with the relevant literature. The challenge of what to leave out of a qualitative data analysis of a transcript never goes away, and some parts of interviews are never suitable for analysis (39).
